# Application of *Arthrospira platensis* for Medicinal Purposes and the Food Industry: A Review of the Literature

**DOI:** 10.3390/life13030845

**Published:** 2023-03-21

**Authors:** Galia Gentscheva, Krastena Nikolova, Veselina Panayotova, Katya Peycheva, Lubomir Makedonski, Pavlo Slavov, Poli Radusheva, Petia Petrova, Ina Yotkovska

**Affiliations:** 1 Department of Chemistry and Biochemistry, Medical University—Pleven, 5800 Pleven, Bulgaria; 2 Department of Physics and Biophysics, Medical University—Varna, 9000 Varna, Bulgaria; 3 Department of Chemistry, Medical University—Varna, 9000 Varna, Bulgaria; 4 Student of Medicine, Medical University—Varna, 9000 Varna, Bulgaria; 5 Institute of Optical Materials and Technologies “Acad. J. Malinowski”, Bulgarian Academy of Sciences, 1113 Sofia, Bulgaria

**Keywords:** *Arthrospira platensis*, medical application, food additive, chemical composition

## Abstract

*Arthrospira platensis is* a filamentous cyanobacterium of the class Cyanophyceae and is the most cultivated photosynthetic prokaryote. It is used in the pharmaceutical sector, medicine and the food industry. It has a rich micro- and macro-element composition, containing proteins, lipids, carbohydrates, essential amino acids, polyunsaturated fatty acids, minerals and raw fibers. It is a commonly used ingredient in food products and nutritional supplements. The wide range of biologically active components determines its diverse pharmacological properties (antioxidant, antidiabetic, antimicrobial, antineoplastic, antitumor, anti-inflammatory, photoprotective, antiviral, etc.). This review summarizes research related to the taxonomy, distribution and chemical composition of *Arthrospira platensis* as well as its potential application in the food and pharmaceutical industries. Attention is drawn to its various medical applications as an antidiabetic and antiobesity agent, with hepatoprotective, antitumor, antimicrobial and antiviral effects as well as regulatory effects on neurodegenerative diseases.

## 1. Introduction

According to the *Handbook of Deterministic Bacteriology*, *Arthrospira platensis* is considered to belong to the oxygenic photosynthetic bacteria that comprise the Cyanobacteria and Prochlorales groups [1]. According to the cylindrical arrangement of the multicellular trichomes (filaments) which is the main morphological feature of the genus, there are two species of filamentous cyanobacteria: *Arthrospira (Spirulina) maxima* and *(Spirulina) platensis* [2]. The two species differ in the helical shape, distribution of gas vacuoles, coil diameter and architecture [3,4]. According to Tomaselli et al. (1993), in *Arthrospira (Spirulina) maxima*, the trichomes have a larger diameter and are not constricted at the cross-walls, while *Arthrospira (Spirulina) platensis* is characterized by short trichomes with a constant diameter of the loose coils. When viewed under SEM, the sheath of *Arthrospira (Spirulina) maxima* appears thicker compared to *Arthrospira (Spirulina) platensis* [5].

This review summarizes research related to the taxonomy, distribution and chemical composition of *Arthrospira platensis* as well as its potential application for medicinal purposes and in the food industry. Due to its rich chemical composition, *Arthrospira platensis* has found various medical application for the prevention of socially significant diseases such as hypertension, obesity, cancer, etc.

*Arthrospira (Spirulina) platensis* naturally inhabits tropical lakes with a high concentration of NaCl and bicarbonates with alkaline waters (pH 11) [6]. Such conditions are limiting for the growth of other microorganisms but allow the cultivation of Spirulina in open reactors [6]. Spirulina can be practically found everywhere: in soil, seawater, freshwater, brackish water, marshes and thermal springs. *Arthrospira platensis* and *Spirulina maxima* thrive in highly alkaline lakes in Africa and Lake Texcoco in Mexico [7].

Recently, there has been an increased interest in the commercial cultivation of *Arthrospira (Spirulina) maxima* and *Arthrospira (Spirulina) platensis*. Often, this takes place in open ponds, where it is not necessary to control parameters such as temperature and light. Along with these advantages, however, there are also a number of disadvantages such as low biomass productivity [8,9] due to the frequent evaporation of water from the reservoir [10] and pollution. An increase in biomass and a higher quality of the product offered is achieved by growing the alga in photobioreactors [11], where the light flux and the amount of carbon dioxide is controlled and the presence of pollutants is reduced. Controlling the temperature conditions in the bioreactors is essential not only for the productivity and biomass of the harvested algae but also for their chemical composition. The latter is notably important for the application of *Spirulina* sp. in medicine, pharmaceutical and food industries. Experiments show that the highest production of 𝛾-linolenic acid (C18:3n-6) was detected at the optimum growth temperatures: 35 and 40 °C for *S. maxima* and 30 °C for *S. platensis* [12]. Solar radiation and temperature not only affected the biomass productivity in *Spirulina* sp. but were also directly related to its improved nutritional characteristics: increased protein, phycocyanin and polyunsaturated fatty acid (mainly γ-linolenic) content [13].

## 2. Chemical Composition

*Arthrospira platensis* are effective producers of bioactive metabolites [14,15,16,17,18,19]. Their protein content accounts for more than 60% of the dry mass [20]. As well as being a rich source of proteins, *Arthrospira platensis* contains appreciable amounts of lipids (7.2% *w*/*w*), carbohydrates (10.3% *w*/*w*), crude fibers (8.5%) and minerals (6.9% *w*/*w*) [19]. Some authors have reported that *Arthrospira platensis* is a source of essential amino acids, polyunsaturated fatty acids, γ-linolenic acid (GLA) and sterols [15,18]. According to [21], *Arthrospira platensis* raw material is rich in vitamins, with the highest content of Niacin B_3_ (14 mg.100 g^−1^), Vit B_6_ (0.8 mg.100 g^−1^), Vit B_12_ (0.32 mg.100 g^−1^), and Vit K (2.2 mg.100 g^−1^). Significant variations in β-carotene content in *Arthrospira* (from 33.5 to 231.6 mg/100 g) were explained by the difference in the geographical region, harvest season and drying method [16,17].

The fatty acid composition of algal lipids comprised mainly of palmitic acid (C16:0, 46%) and linoleic acid (C18:2n-6, 17.4%) with a lower content of γ-linolenic acid (C18:3n-6), palmitoleic acid (C16:1n-7), oleic acid (C18:1n-9) and myristic acid (C14:0) [21]. Analysis of the amino acid composition showed that *Spirulina* is characterized by a high quantity of amino acids: glutamate (8.15%), aspartate (5.34%), alanine (4.54%), leucine (4.84%), arginine (3.96%), valine (3.34%), glycine (3%), tyrosine (2.58%) and proline (2.15%) [22]. According to [23,24], the metabolizable energy after consuming *Spirulina* varies from 2500 to 3290 kcal/kg.

According to [1], *Arthrospira platensis* powder has a rich mineral composition, with the highest total content being potassium (1400 mg.100 g^−1^), sodium (900 mg.100 g^−1^), phosphorus (800 mg.100 g^−1^), calcium (700 mg.100 g^−1^), magnesium (400 mg.100 g^−1^), and iron (100 mg.100 g^−1^). Furthermore, the algae are rich in selenium and cyanobacterial selenium, which was found to exist as selenite (2%) and selenomethionine (18%) [25]. Sukumaran et al. stated that high levels of calcium and magnesium were observed in *Arthrospira platensis* cultured in salt water compared to the one in freshwater [26]. In contrast to these elements, *Arthrospira platensis* has a higher content of the remaining microelements when cultivated in a freshwater environment [26]. Campanella et al. noted a high content of iron in products from the seaweed grown in Cuba [27]. They point out that the experiment with rats fed with fresh *Arthrospira platensis* showed a good absorption of the specified element and also that the Fe content in the algae was much higher than in many other plants. Furthermore, different forms of storage can also affect the quantity and quality of bioactive substances. Papalia et al. [28] evaluated different effects of freezing, oven drying and freeze drying on the chemical composition of *Arthrospira platensis.*

Table 1 illustrates some significant antioxidant biomolecules in *Arthrospira platensis.* As it can be seen, *Arthrospira platensis* is a rich source of flavonoids, vitamins and pigments [28,29,30].

The dietary fiber in *Arthrospira platensis* has been shown to promote the growth of beneficial microorganisms in the gastrointestinal tract (*Lactobacillus casei*, *L. acidophilus*, *Streptococcus* and *Bifidobacterium* spp.) and to reduce populations of harmful ones [31]. The polyphenolic components in *Arthrospira platensis* have a proven antidiabetic effect. The polyphenol-rich butanol extract of the alga is a potent α-glucoside inhibitor (IC_50_ 23 µg/mL). Intestinal α-glucosidases inhibitors are important in controlling diabetes because they lower postprandial blood glucose levels [32]. The most interesting of the *Arthrospira platensis* pigments is the blue pigment phycocyanin. The authors of some studies note that it can exhibit an antidiabetic effect through the inhibition of α-amylase and α-glucosidase [33]. Hamsters, which receive phycocyanin, were observed to reduce plasma total cholesterol and LDL cholesterol, especially when administered together with selenium [34].

## 3. Possible Health Risks

Cyanobacteria are primary producers of toxins (anatoxins, saxitoxin, and related analogs). *Arthrospira platensis* does not produce anatoxin-𝛼, while *Spirulina maxima* is a potential producer [35]. The latter toxin may pose a health risk because it is an acetylcholine receptor agonist causing paralysis, muscle contractions, and respiratory failure [36]. Microcystin production is particularly dangerous in strains of the algae when harvested from open lakes [37]. It is easier to monitor the quality of *Arthrospira platensis* from closed photobioreactors.

*Arthrospira platensis* is known to absorb Pb from the surrounding aquatic bodies and is used for purification purposes. Siva et al. note that the latter is undesirable in the use of the algae in food and pharmaceutical technology [38]. The European Commission established a maximum permissible limit of 3 mg/kg for lead (European Commission, 2006) in food supplements. The European Commission has not yet imposed limits for pesticides but keeps special attention to their control when using *Arthrospira platensis* for food and pharmaceutical purposes.

## 4. Spirulina as Food Additive in Nutritional Foods

Considering the rich chemical composition of algae, it is not surprising that they are used as nutritional components in various forms: powder, tablets, extracts or supplements. Adding *Arthrospira platensis* extract or phycocyanin (extracted from *Arthrospira platensis*) to yogurt or green tea increases their nutritional values [39].

The addition of *Arthrospira platensis* powder to bakery products improves their nutritional value due to the presence of higher amounts of vitamins and trace elements [40]. The results revealed in terms of texture, expansion coefficient, centesimal composition and sensory acceptance the feasibility of this enrichment without affecting in a significant manner the typical characteristics of the products, including satisfactory sensory acceptance [40]. *Spirulina* supplementation has a stimulating effect during the fermentation process and storage of beneficial bacteria such as *Lactobacillus acidophilus* (*Lactobacillus gallinarum*), *Lactobacillus bulgaricus* [41,42,43] and *Lactobacillus casei* (*Lacticaseibacillus rhamnosus*) [44]. The result is an improvement in the quality of fermented milk products [44,45]. Furthermore, the presence of phycocyanin at different concentrations (2, 4, and 8%) in Spirulina-enriched yogurt results in an increase in pH values from 4.4 to 4.74, 4.80, and 4.92, respectively [39]. Several articles have reported that the addition of 1.0 and 0.5% *Arthrospira platensis* leads to an increase in the antioxidant activity of yogurt by 200% and 110%, respectively [46,47]. The high tocopherol and phenolic content in *Arthrospira platensis*, as well as the presence of phycocyanin, leads to a substantial increase in the antioxidant activity of processed cheese in the presence of 3% of the specified algae [48].

The addition of *Spirulina* from 1% to 2% to the ice cream has a beneficial effect on its texture and melting point [49]. The addition of 2% inulin and 1% *Arthrospira platensis* into the ice cream preparation showed the same taste qualities as traditional ice cream but with less fat (50%) and sugar (25%) [50]. *Arthrospira platensis* powder added to fruit or vegetable juice increases exercise endurance and improves metabolism in adults [51]. A study performed by [52] aimed to feed a bee colony with *Arthrospira platensis* in its natural and usual life cycle and as a result of the application of this special feeding method to obtain a new green-colored functional honey produced by bees containing *Spirulina* and sugar-free. This seaweed honey shows high antioxidant activity and significant content of caffeic acid, kaempferol, ω−3, and ω−6 fatty acids [52].

Due to its high nutritional value, essential element content, protein composition, amino acids and essential fatty acids, *Arthrospira platensis* has found an application as a component of various food products (Table 2).

## 5. Medical Application of *Arthrospira platensis*

**Figure 1:**
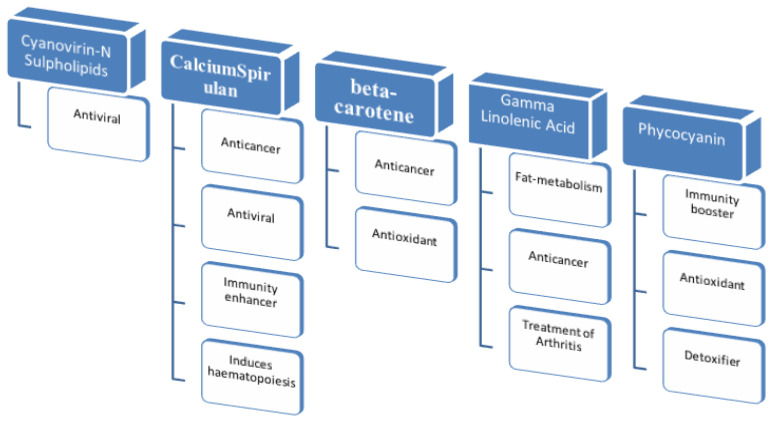
Medical applications of the main biomolecules in *Arthrospira platensis*.

*Arthrospira platensis*, besides its nutritional value and rich mineral composition, has specific therapeutic immunomodulating, immunostimulating, biomodulating, antitumor, and metabolic effects [62]. Therefore, in the second part of this review, a special focus is placed on the medical applications of *Arthrospira platensis* and the possibilities for the development of various nutritional supplements. Figure 1 represents the therapeutically important components of *Arthrospira platensis* and its main medical uses.

### 5.1. Antimicrobial and Antiviral Effects

Ethanol, methanol and aqueous extracts of the algal plant *Arthrospira platensis* were evaluated for their antimicrobial activity against four types of Gram-positive bacteria, namely *Staphylococcus aureus*, *Streptococcus pneumoniae*, *Bacillus cereus* and *Enterococcus faecalis*. According to [63], the aqueous extract showed no antimicrobial activity, the ethanolic extract successfully fought *Enterococcus faecalis* and *Staphylococcus aureus,* while the methanolic extract had the strongest antimicrobial effect on *Streptococcus pneumoniae* and *Bacillus cereus*. In addition, the *Arthrospira platensis* has been shown to have significant antimicrobial activity against strains of *Vibrio parahaemolyticus*, *Vibrio anguillarum*, *Vibrio splendidus*, *Vibrio scophthalmi*, *Vibrio alginolyticus* and *Vibrio lentus* [64]. Phycocyanin extracted and purified from the algae can be used to combat drug resistance, as it significantly inhibits drug-resistant bacteria such as *E. coli*, *Klebsiella pneumoniae*, *Pseudomonas aeruginosa*, and *S. aureus* [65].

There is evidence that *Arthrospira platensis* has antibacterial and antifungal activity [66]. To establish the maximum antifungal effect of the algae, extracts with methanol, hexane, and acetone were tested. The methanolic extract has been shown to have the highest antioxidant and antimicrobial activity [67,68]. It has been found that due to its enhanced antimicrobial properties, the algae are extremely suitable in food products as a preservative and can lead to an increase in their shelf life [69]. In addition to its antimicrobial activity, *Arthrospira platensis* exhibits broad-spectrum antiviral activity because of its high content of polysaccharides, inhibiting the replication of enveloped viruses such as herpes simplex virus, influenza virus, measles virus, mumps virus, and HIV-1 [70,71]. The active components in the algal extracts are sulfated polysaccharide such as calcium spirulan (Ca-SP) [72].

There are a number of studies attesting to a significant improvement in the parameters of the immune system of HIV patients after taking *Arthrospira platensis* [70]. Other authors noted a reduction in viral load after six months of *Arthrospira platensis* intake in combination with a balanced diet [71]. Analyzing blood cells of volunteers in Japan with pre- and post-oral administration of hot water extract of *Spirulina* was performed [73]. A beneficial effect on markers of the immune system was indicated, and an ability to inhibit carcinogenesis was confirmed [74]. Lobner et al. extracted a high molecular weight Immulina polysaccharide from *Arthrospira platensis*, which has a positive effect on the immune system [75]. A study in Denmark on experimental animals and humans testified that Immulina was highly active against *Candida albicans* and *tetanus* [76]. Another component with a powerful immunostimulating effect in the blue–green algae is phycocyanin. Experimental animals taking phycocyanin show resistance to various infectious diseases [77]. In addition, this compound shows properties to reduce allergic inflammation by suppressing the antigen-specific IgE antibody [78]. *Arthrospira platensis* activates 164 functions of macrophages, phagocytosis, and the primary immune response of the cell [79]. Baojiang et al. proved that algae’s polysaccharides affect and improve specific and non-specific cellular immunity [80].

In recent years, numerous studies have been published on the impact of cyanobacteria on COVID-19. Scientists from India, Japan, China, and the USA are studying this superfood with the aim of using it against the viral load or prevent this disease [62]. Hernández-Corona et al. reported high antiviral activity in extracts of *S. maxima* prepared from methanol–water (3:1) probably because the extracts have been shown to contain sulfated polysaccharides that significantly prevent several viruses from attaching to the host cell [81]. They do not kill the virus but prevent it from entering the cells of a healthy organism [82]. Sayda et al. demonstrated that water–methanol extracts of *Arthrospira platensis* were effective against adenovirus type 40 and reduced infection by 23% to 50% using non-toxic extract concentrations of 2 mg/mL [83]. Based on the literature data, it can be concluded that *Arthrospira platensis* can be used as a food and nutritional supplement or drug of great clinical interest due to its antiviral activity and body’s immune response against pathogens. Further various medical applications of seaweed are of concern—for example, it can be used against anemia, diabetes, cardiovascular diseases, malignant formations, etc.

### 5.2. Application for Allergies and Rhinitis

The anti-inflammatory property of *Spirulina* is related to the inhibition of histamine release from mast cells. Patients with allergic rhinitis taking *Arthrospira platensis* powder for around 12 weeks showed reduced levels of IL-4 by 32%, which was probably due to the protective effect of microalgae toward allergic rhinitis [84]. A similar study was performed in Turkey on patients with allergic rhinitis and found that *Arthrospira platensis* consumption significantly improved the symptoms and physical findings compared with placebo, including nasal discharge, sneezing, nasal congestion and itching [85]. In addition to its rich mineral content, vitamin and amino acids composition, antiviral and anti-inflammatory action, *Arthrospira platensis* has also a regulatory effect on cholesterol level, oxidative stress, mitochondrial dysfunction, and neurodegenerative disorders [86,87].

### 5.3. Regulatory Effect on Neurodegenerative Disorders and Physical Endurance

**Figure 2:**
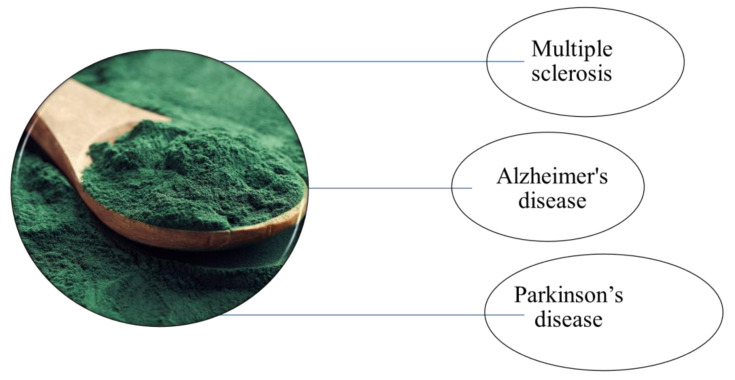
Application of *Arthrospira platensis* in the treatment of neurodegenerative diseases.

A number of studies on the neuronal loss have been conducted with experimental animals. Neuronal loss is associated with the overproduction of free radicals, which normally occurs during cell metabolism. When the production of free radicals exceeds the natural antioxidant systems of the organism, it can cause cell damage and subsequent cell death. Pérez-Juárez et al. had demonstrated that even a single intake of *Arthrospira platensis* reduced neuronal death in experimental animals [88]. It was previously reported that *Arthrospira platensis* reduces oxidative stress in the hippocampus and protects against the damaging neurobehavioral effects of kainic acid [89]. However, it is not known if these effects are related to a reduction in neuronal damage in the hippocampus [88]. During the adolescent period (PNDs 30–40), rats were subjected to restraint stress (2 h/day for 10 days). Then, the animals were subjected to 15 days’ treatment (PNDs 41–55) with *Arthrospira platensis* (200 mg/kg/day) followed by biochemical, molecular, and morphological assessments in the basolateral amygdala. The findings of this study provide important evidence that *Arthrospira platensis* as a non-pharmacological intervention during the adolescent period can protect against chronic stress-induced neuroanatomical biochemical, and molecular deficits in adulthood, and thus, reduce stress-related disorders [90]. In their work, [91] demonstrate that the phytosterols of marine algae (ex: fucosterol) have been investigated for many health benefits, including antidiabetes, anti-obesity, anti-Alzheimer’s, antiaging, anticancer, and hepatoprotection, among many others, which are attributed to their antioxidant, anti-inflammatory, immunomodulatory and cholesterol-lowering properties, indicating their potentiality as therapeutic leads. Some authors have reported that *Arthrospira platensis* supports the resistance to the oxidative aspect of microglial cell activity and fights against memory loss [92,93]. By reducing oxidative stress and increasing the amount of substances with antioxidant properties in the body, *Arthrospira platensis* exerts a neuroprotective effect on the body [94,95]. Ref. [96] reported that selenium-enriched *Arthrospira platensis* significantly improved neuronal viability (from 57.2% to 94.5%) and inhibited apoptosis in oxygen glucose deprivation (OGD)-treated primary neurons (from 45.6% to 6.3%), which was followed by improved neuronal morphology and caspases activation. The selenium-enriched *Arthrospira platensis* effectively suppressed OGD-induced DNA damage by inhibiting reactive oxygen species (ROS) accumulation in neurons (from 225.6% to 106.3%) [96]. Ref. [97] studied the role of phycocyanin in improving cognitive dysfunction in rats subjected to intracerebroventricular induction of streptozotocin (STZ) and demonstrated the immense potential of phycocyanin in attenuating STZ-induced cognitive decline. There is evidence of the application of blue–green algae for the treatment of multiple sclerosis (MS). Studies on rats suffering from MS were treated with C-Phycocyanin. The authors noted that the experimental animals have firm, albeit crumpled, myelin sheaths with no signs of axonal disintegration, so they concluded that C-Phycocyanin protects axons from demyelination [98,99]. Due to its strong antioxidant effect, *Arthrospira platensis* may be used for the development of anti-inflammatory pharmacological products, which has an effect on the brain parenchyma and further for the prevention and treatment of neurological disorders such as Alzheimer’s disease (AD), Parkinson’s disease (PD) and MS (Figure 2).

The intake of *Arthrospira platensis* contributes to stress resistance and mental strain and increases the body’s resistance to physical fatigue as a result of sports activity. After 1 week, a 3 g/day dose of *Arthrospira platensis* produced a small but statistically significant increase in exercise output [100]. A double-blind, randomized, crossover, controlled trial was designed to evaluate the independent and synergistic effects of *Arthrospira platensis* supplementation (4.5 g/day) with or without performing a physical exercise program on the blood lipids and body mass index (BMI) of overweight and obese men. Comparing the final vs. the initial values, BMI, total cholesterol, triglycerides and low-density lipoprotein cholesterol were decreased. *Arthrospira platensis* supplementation enhances the hypolipidemic effect of a systematic physical exercise program in men with excess body weight and dyslipidemia [101]. Kalafati et al. reported that *Spirulina* supplementation induced a significant increase in exercise performance, fat oxidation, and reduced glutathione (GSH) concentration and attenuated the exercise-induced increase in lipid peroxidation in physically active men [102].

### 5.4. Antidiabetic, Anti-Obesity, Anti-Hypertensive and Hepatoprotective Effects

Several clinical and preclinical trials have been conducted to test the benefits of *Arthrospira platensis* on weight loss [103]. Green algae have been shown to regulate lipid and carbohydrate metabolism [104] and to have cholesterol-lowering effects [105]. Phycocyanin in *Arthrospira platensis* suppresses oxidative stress and leads to anti-inflammatory and insulin-sensitizing effects [106,107,108]. A parallel pilot study of 4.5 g administration of *Arthrospira platensis* or placebo for 12 weeks in 16 patients with systemic arterial hypertension (SAH) undergoing treatment with angiotensin-converting enzyme (ACE) inhibitors was performed to assess the effects on endothelial damage and oxidative stress indicators. The results showed a statistically significant decrease in systolic blood pressure, sVCAM-1, sE-selectin and endothelin-1 levels as well as an increase in glutathione peroxidase activity and oxidized glutathione levels [109]. Miczke et al. reported in their study that three months of regular consumption of *Arthrospira platensis* not only improves BMI and weight but also results in improvements in blood pressure and endothelial function in overweight patients with hypertension but lacking evidence of cardiovascular disease [110]. In the process of investigating the hypolipidemic effects of *Arthrospira platensis*, Han et al. found that the aqueous extract of it may inhibit the intestinal absorption of dietary fat by inhibiting pancreatic lipase activity [111]. Khan et al. studied whether *Spirulina* could serve as a cardioprotective agent during doxorubicin-induced cardiotoxicity (DOX) treatment in a mouse model [112]. A similar effect was observed when taking pure phycocyanin extracted from the cyanobacteria. Khan et al. investigated the cardioprotective effect of C-phycocyanin (PC) (an antioxidant biliprotein pigment of a blue–green algae *Arthrospira platensis*) against ischemia–reperfusion (I/R)-induced myocardial injury and show that PC attenuated I/R-induced cardiac dysfunction through its antioxidant and antiapoptotic actions [112].

Yousefi et al. studied 52 obese patients with a body mass index (BMI) > 25–40 kg/m^2^ who were divided into two groups: the first group took 2 g *Arthrospira platensis* per day with a restricted caloric diet, and the second group was a placebo consisting of a restricted calorie diet for 12 weeks [113]. After a few weeks, participants in the *Arthrospira platensis* group had significantly lower BMI, waist circumference, body fat, triglycerides and high-sensitivity C reactive protein levels. The consumption of spirulina leads to a decrease in blood lipid levels and an improvement in the immune and antioxidant response in obese people [114].

*Arthrospira platensis* has shown hepatoprotective activities due to the presence of large amounts of antioxidant compounds. Fatty acids, mineral composition, vitamins E and C, and phenolic compounds provide protection and strengthen liver functions [115]. Algae-derived C-phycocyanin successfully reduced lipid peroxidation in liver microsomes from CCl4-intoxicated rats [116].

### 5.5. Effect on Anemia and Reactive Oxygen Radicals

*Arthrospira platensis* has an exceptionally high content of vitamin B_12_. Therefore, some authors suggest that the use of spirulina dietary supplements may ameliorate anemia [117,118]. A study was conducted among elderly people with anemia with no history of major chronic diseases. Participants took 6 tablets of 500 mg *Arthrospira platensis* daily for 12 weeks. The authors suggest that the intake of the supplement favorably affects anemia and immune dysfunction [117].

A significant increase in hemoglobin levels was observed when taking 1–2 g per day for six to seven weeks [119]. Taking *Arthrospira platensis* capsules improved the hematological parameters and intellectual status of girls aged between 7 and 9 years [120]. *Arthrospira platensis* intake by pregnant women has been shown to help prevent hemoglobinemia. The authors mention that the inclusion of the alga in the menu of this group has a more favorable effect on the general condition than the intake of iron and folic acid [121].

Supplementation diets with *Arthrospira platensis* could improve the nutritional status of children who were underweight [122]. A prospective study was conducted in the Democratic Republic of the Congo (DRC) in malnourished children under five. Children were divided into two groups: (1) the control group with children consuming food rich in vitamins and microelements and (2) the intervention group of children consuming the same diet but with the addition of 5 g *Arthrospira platensis* twice a day. A significant increase in hemoglobin and hematocrit values was observed in children in the intervention group [122].

*Arthrospira platensis* contains a number of phenolic compounds, carotenoids and fatty acids which have a powerful antioxidant effect and protect cells from the harmful effects of reactive oxygen species. Ref. [123] demonstrated that the dietary supplementation of *Arthrospira platensis* prevents oxidative stress and improves vascular reactivity in animals with endothelial dysfunction. Phycocyanins, chlorophyll, β-carotene, and other carotenoids, vitamins and minerals in *Arthrospira platensis* also mediate the antioxidant and protective effects [30,124]. Chlorophyll and its derivatives are used widely in pharmaceutical products. Chlorophyll has been found to accelerate wound healing and prevent bacteria growth. Due to the high amounts of chlorophyll, orally administered *Arthrospira platensis* biomass and *Arthrospira platensis* extract exerts gastro-protective effects [125,126].

### 5.6. Antitumor Properties

Beta-carotene is a natural compound that may inhibit the formation, growth, and development of tumor tissue. In vitro studies showed that *Arthrospira platensis* aqueous extracts may inhibit the proliferation, motility, and invasion of colorectal cancer cell lines. Ramakrishnan demonstrated that chloroform extract of crude *Arthrospira platensis* and *Chlorella vulgaris* inhibited the viability of breast cancer cells in vitro [127]. Extracted *Arthrospira platensis* polysaccharides with selenium nanoparticles may be a potential candidate against human cancers as a chemopreventive and chemotherapeutic agent [128] showing a strong antiproliferative factor on human melanoma and breast adenocarcinoma cells [129].

Schwartz and Shklar studied the effect of phycotene extract of *Arthrospira platensis* and Dunaliella algae on tumor regression [130]. Total tumor regression was found in 30% and partial tumor regression was found in the remaining 70% of the animals after four weeks. The phycocyanin isolated from *Arthrospira platensis* showed anticancer activity against squamous cell carcinoma in hamsters [130]. Microscopic sections of the buccal pouch of hamsters supplemented with *Arthrospira platensis* and Dunaliella algae extracts showed localized areas of dysplasia and early carcinoma undergoing destructions [131]. There was no evidence of toxicity in any of the animals receiving the algae extracts [130,131].

*Arthrospira platensis fusiformis* also exhibits chemopreventive potential in humans with leukoplakia [132]. The authors observed a partial regression of tumor lesions that developed on the inside of the cheek in 45% of patients and complete regression in 7% of patients [132].

Phycocyanin (160 mg L^−1^) from *Arthrospira platensis* significantly inhibited the growth of human chronic myelogenous leukemia-blast crisis K562 cells in a dose-dependent manner [133].

### 5.7. Arthrospira platensis and Eye Health

*Arthrospira platensis* is rich in β-carotene, but it contains other important carotenoids such as zeaxanthin and β-cryptoxanthin, myxoxanthophyll and echinenone [134]. Zeaxanthin, a xanthophyll that exists in human eyes, plays a role in decreasing the probability for cataracts and age-related macular degeneration. Dried *Arthrospira platensis* powder as a dietary supplement increases the serum zeaxanthin levels in humans [135].

### 5.8. Antitoxic Activity

*Arthrospira platensis* exhibited a significant protective effect against lead-induced oxidative stress in the kidney of newborn rats and prevented oxidative damage induced by lead in the renal tissue [136]. The modulatory effect of *Arthrospira platensis* on lead toxicity may be due its high content of flavonoids which are powerful antioxidants, β-carotene, iron, γ-linolenic acid, other carotenoids, phycocyanins and vitamins [136,137,138]. Ebaid et al. demonstrated the protective effects of *Arthrospira platensis* against nano-CuO-induced hepatotoxicity in albino rats [138]. The oral administration of *Arthrospira platensis* solution could exhibit a protective activity against hepatotoxicity caused by heavy metals nanoparticles. *Arthrospira platensis* has been found to positively affect the levels of antioxidant enzymes and markers of oxidative stress against deltamethrin toxicity [138].

## 6. Conclusions

*Arthrospira platensis* contains many biologically active hydrophilic and lipophilic substances that have therapeutic effects on tissues, blood cells and organs. Accumulated scientific evidences for the beneficial effects of *Arthrospira platensis* on human health has been demonstrated in animal and human studies. The pharmacological action reported in them included immunomodulation, antioxidant activity, antiviral effect, applications in allergies and rhinitis, effects against diabetes, hypertension and hyperlipidemia.

## Figures and Tables

**Table 1:** Content of significant antioxidant biomolecules in *Arthrospira platensis* [28,29,30].

Item	Content
Total phenols, (μmol GAE/g) in carotenoids extracts	1.3–6.4
Total flavonoids (μmol QE/g) in carotenoids extracts	12.9–26.6
Total carotenoids (mg/g for dry weight)	0.28–4.43
All-trans-β-carotene (mg/g for dry weight)	0.02–2.3
Chlorophyll a (mg/g for dry weight)	2.7–10.8
C-Phycocyanin (mg/g for dry weight)	94.9–251.2

**Table 2:** *Arthrospira platensis* as ingredient in food products.

Type of Food Products	Concentration of *Arthrospira platensis*	References
Cookies	(10–15)%	[53]
Snack	(0–12.5)%	[54]
Baby food formulation	(0–7.5)%	[55]
Pasta	(0–1)%	[56]
	(1–15)%	[57]
Ice cream	5%	[58]
Low fat probiotic yogurt	(0.1–1)%	[59]
Yogurt	0.5% and 1%	[60]
Functional Yogurt	(0.1–0.5)%	[61]

## Data Availability

Datasets from the time of this study are available from the respective authors upon reasonable request.
